# Evaluating the therapeutic impact of early multimodal rehabilitation in hemiplegic stroke patients: A quasi-experimental study

**DOI:** 10.1038/s41598-026-54146-3

**Published:** 2026-05-20

**Authors:** Jasmin Behera, Mamata Swain, Jhunilata Pradhan, Sasmita Das

**Affiliations:** 1 Department of Medical Surgical Nursing, Sum Nursing College, S‘O’A Deemed to be University, Bhubaneswar, India; 2 Sum Nursing College, SOA University, Bhubaneswar, Odisha India

**Keywords:** Early rehabilitation therapy, Stroke severity, Cognitive functions, Motor functions, Hemiplegia patients, Neuroscience, Health care

## Abstract

Early rehabilitation following stroke has emerged as a pivotal strategy to accelerate recovery and enhance functional outcomes in hemiplegic patients. Despite supporting evidence, the integration of structured rehabilitation protocols in critical care settings remains inconsistent, particularly in resource-limited environments. This study aimed to evaluate the effectiveness of early rehabilitation therapy on stroke severity, cognitive function, and motor performance among hemiplegic stroke patients admitted to a critical care unit. A quasi-experimental design was employed. Fifty-two participants were recruited and evenly assigned to experimental and control groups. The experimental group received a structured multimodal rehabilitation program comprising motor retraining, cognitive stimulation, and task-specific functional practice initiated within 72 h of stroke onset. The control group received standard post-stroke care. Outcomes were assessed using the NIH Stroke Scale (NIHSS), Mini-Mental State Examination (MMSE), and Fugl-Meyer Assessment (FMA). After four weeks, the experimental group demonstrated significantly improved outcomes: 92.3% achieved minor stroke severity, 80.8% attained normal cognitive scores, and 46.2% exhibited only slight dyscoordination in motor tasks. Large effect sizes across all outcome measures confirmed the clinical relevance of early intervention. The findings underscore the importance of timely, interdisciplinary rehabilitation in enhancing neurological recovery after stroke. Critical care nurses play an essential role in promoting early mobilization, integrating cognitive and motor rehabilitation strategies, and adopting standardized protocols to improve functional independence and reduce long-term disability post-stroke.

## Introduction

Stroke remains one of the leading causes of mortality and long-term disability worldwide. Globally, it contributes substantially to functional dependence and reduced quality of life among survivors^1^. Hemiplegia, characterized by paralysis or severe weakness on one side of the body, is among the most common and disabling consequences of stroke, significantly impairing mobility, self-care, communication, and participation in social and occupational roles^2^. Beyond motor deficits, stroke survivors frequently experience cognitive impairments including deficits in attention, memory, processing speed, and executive functioning—which further compromise functional recovery and independence^3,4^.

In low- and middle-income countries such as India, the burden of stroke is increasing due to demographic transition, rising prevalence of vascular risk factors, and limited access to structured rehabilitation services^5^. Stroke survivors in resource-constrained settings often experience delayed or fragmented rehabilitation, contributing to prolonged disability and increased caregiver burden^6^. The long-term physical, emotional, and financial consequences of hemiplegia place substantial strain on families and healthcare systems, emphasizing the need for cost-effective and evidence-based rehabilitation models^7^. Stroke recovery is strongly influenced by neuroplasticity, the brain’s ability to reorganize neural pathways following injury. Experimental and clinical evidence suggests that the early post-stroke period represents a critical window during which the brain demonstrates heightened responsiveness to therapeutic stimulation^8,9^. Interventions initiated during this phase may enhance cortical reorganization and optimize functional recovery outcomes. Consequently, early rehabilitation has emerged as a key principle in contemporary stroke management guidelines^2,10^.

Traditionally, stroke rehabilitation has predominantly focused on motor recovery through early mobilization, strength training, balance exercises, and task-specific practice^1,11^. While these interventions have demonstrated effectiveness in improving motor function, cognitive impairments are frequently under-addressed during the acute inpatient phase despite their strong association with poor long-term functional outcomes^3,12^. Studies have shown that cognitive deficits independently predict reduced activities of daily living, decreased participation, and lower quality of life among stroke survivors^4^. Emerging evidence indicates that structured cognitive rehabilitation interventions targeting memory, attention, and executive function can improve neuropsychological performance and functional outcomes in subacute stroke populations^13^. However, most previous investigations have examined motor and cognitive rehabilitation strategies independently rather than integrating them into a unified therapeutic framework. Furthermore, many randomized controlled trials evaluating early rehabilitation have been conducted in high-resource settings, limiting generalizability to tertiary care centers in developing countries where rehabilitation delivery systems differ significantly^10,11^. Limited evidence exists examining the combined short-term effects of early integrated motor and cognitive rehabilitation initiated during acute hospitalization using standardized neurological outcome measures.

Early multimodal rehabilitation refers to a structured, interdisciplinary therapeutic approach initiated during the acute phase of stroke typically within the first 24 to 72 h following medical stabilization that simultaneously targets multiple domains of recovery, including motor retraining, cognitive stimulation, sensory activation, and task-oriented functional practice^10,14^. Unlike conventional post-stroke care, which primarily emphasizes prevention of complications and passive range-of-motion exercises, early multimodal rehabilitation integrates coordinated motor and cognitive interventions designed to harness the window of enhanced neuroplasticity during early recovery^8,9^. The theoretical basis of this approach lies in the principle that simultaneous activation of motor and cognitive neural networks enhances cortical reorganization and strengthens functional neural connectivity^15^. Task-specific motor practice stimulates sensorimotor cortical pathways, while cognitive exercises targeting executive function, attention, and memory engage prefrontal and associative networks. The integration of these domains may promote synergistic neural recovery, improve task performance, and accelerate functional independence^13,15^.

Several studies have demonstrated that early mobilization and task-oriented motor training improve functional recovery and reduce disability after stroke^1,11^. Similarly, cognitive rehabilitation programs have been shown to enhance attention, memory, and executive functioning in stroke survivors^13^. However, relatively few studies have systematically combined structured cognitive rehabilitation with progressive motor training during the acute inpatient phase. Moreover, limited research from resource-constrained tertiary care settings has simultaneously evaluated changes in stroke severity, cognitive performance, and motor recovery using standardized assessment tools such as the National Institutes of Health Stroke Scale (NIHSS)^16^, Mini-Mental State Examination (MMSE)^17^, and Fugl-Meyer Assessment (FMA)^18^. Therefore, there remains a need to evaluate a structured early multimodal rehabilitation protocol integrating motor and cognitive components within the acute post-stroke phase and to examine its short-term therapeutic impact using comprehensive neurological outcome measures. Addressing this gap may contribute valuable contextual evidence for developing multidisciplinary rehabilitation protocols tailored to stroke survivors in similar healthcare settings.

## Methods

### Ethical Considerations

#### Ethical approval

was obtained from the Institutional Review Board of Institute of medical sciences (IMS) and SUM hospital, Bhubaneswar, Odisha (Letter no- Ref.no/IEC/IMS.SH/SOA/2024/882). The study protocol adhered to ethical standards for human research, with emphasis on participant safety, autonomy, and confidentiality. Written informed consent was obtained from all participants in accordance with the Declaration of Helsinki. No financial or coercive incentives were offered. Data were anonymized and securely stored. Both control and experimental groups received appropriate clinical oversight to ensure safety and dignity throughout the study.

### Study design and approach

This study employed a quasi-experimental pre-test–post-test control group design to evaluate the effectiveness of early multimodal rehabilitation on neurological, cognitive, and motor recovery in hemiplegic stroke patients. The study was conducted over four weeks at a tertiary care hospital in Bhubaneswar, Odisha, India.

### Sample size estimation

A priori sample size estimation was performed using G*Power software version 3.1 for repeated-measures ANOVA with within–between interaction. Assuming a medium effect size (f = 0.25), an alpha level of 0.05, statistical power of 0.80, two groups, and four measurement time points (baseline and weekly assessments over four weeks), the minimum required sample size was calculated as 48 participants. The anticipated effect size was derived from previous early rehabilitation studies demonstrating moderate improvements in neurological severity and motor recovery following structured interventions^16,17^. To account for potential attrition, the final sample size was increased to 52 participants, with 26 participants allocated to each group.

### Sampling technique and allocation procedure

Participants were recruited using purposive sampling based on predefined eligibility criteria. After obtaining informed consent, eligible participants were randomly allocated to either the experimental group (*n* = 26) or the control group (*n* = 26) using a simple randomization technique. Each participant was assigned a unique identification number written on identical opaque slips. A blinded third party conducted the random draw to ensure allocation concealment and minimize selection bias.

### Inclusion criteria

Inclusion criteria consisted of adult patients (aged 25–72 years) with first-ever ischemic or hemorrhagic stroke resulting in hemiplegia, who were medically stable and within 72 h to 14 days of stroke onset. To ensure clinical homogeneity and safety for early rehabilitation, only patients with moderate neurological impairment were included, defined as a National Institutes of Health Stroke Scale (NIHSS) score between 5 and 20, based on established stroke severity classifications^16^. Participants were further required to demonstrate mild to moderate cognitive impairment, indicated by a Mini-Mental State Examination (MMSE) score between 10 and 23 according to standard interpretative guidelines^17^, and marked motor impairment defined by a Fugl-Meyer Assessment (FMA) motor domain score between 50 and 84, as described in the original FMA classification system^18^. These criteria were applied to ensure that participants were clinically stable, capable of actively engaging in structured rehabilitation interventions, and sufficiently homogeneous to permit valid comparison of outcomes.

### Exclusion criteria

Patients with severe stroke (NIHSS > 20), severe cognitive impairment (MMSE < 10), profound motor impairment (FMA < 50), recurrent stroke, unstable medical condition, aphasia preventing cognitive assessment, or other neurological disorders were excluded from the study. The application of these exclusion criteria ensured participant safety, enhanced clinical homogeneity, and strengthened internal validity by selecting individuals who were medically stable and capable of actively participating in early multimodal rehabilitation interventions.

### Data collection procedure and tools

Demographic and clinical baseline data were collected using a self-structured socio-demographic and clinical questionnaire. Three standardized assessment tools were used to measure study outcomes.Stroke severity was assessed using the NIHSS^16^, cognitive function by MMSE^17^, and motor function by the Fugl-Meyer Assessment (motor domain)^18^, all of which have established reliability and validity.

The **National Institutes of Health Stroke Scale (NIHSS)** is a standardized neurological assessment tool used to quantify stroke severity in clinical and research settings^16^. The scale comprises 15 items assessing domains such as level of consciousness, gaze, visual fields, motor strength, sensory function, language, and neglect, with total scores ranging from 0 to 42. Stroke severity is categorized as minor (0–4), moderate (5–15), moderate-to-severe (16–20), and severe (21–42) according to the original classification by Brott et al.^16^. In the present study, content validity of the NIHSS was re-evaluated by a panel of five experts in neurology and rehabilitation nursing, yielding a Content Validity Index (CVI) of 0.92. Inter-rater reliability was assessed in a pilot sample of stroke patients, demonstrating high agreement among evaluators.

The **Mini-Mental State Examination (MMSE)** is a 30-point standardized instrument used to assess global cognitive function across domains including orientation, memory, attention, language, and visuospatial skills^17^. Scores range from 0 to 30, with established interpretative thresholds: 24–30 (normal cognition), 19–23 (mild impairment), 10–18 (moderate impairment), and < 10 (severe impairment)^17^. An unauthorized version of the English MMSE was used by the study team without permission, however this has now been rectified with PAR. The MMSE is a copyrighted instrument and may not be used or reproduced in whole or in part, in any form or language, or by any means without written permission of PAR. For the present study, content validity was established through expert review, resulting in a CVI of 0.95. Reliability testing conducted during pilot administration demonstrated satisfactory internal consistency and stability over time.

The **Fugl-Meyer Assessment (FMA)** is a performance-based scale widely used to evaluate motor recovery following stroke^18^. The motor function subscale, utilized in this study, has a total score range of 0–100, with higher scores indicating better motor performance. Motor impairment categories were defined according to the original Fugl-Meyer framework^18^. In this study, the instrument underwent expert validation, yielding a Content Validity Index (CVI) of 0.97. Inter-rater reliability testing among trained assessors demonstrated excellent agreement, confirming the tool’s suitability for use in the study population.

### Early multimodal rehabilitation intervention

The Early Multimodal Rehabilitation (EMR) program was implemented (table-1) for duration of four weeks in the experimental group. Participants received three sessions per week, with each session lasting approximately 45–60 min, resulting in a total of 12 supervised sessions during the intervention period.

Each session was structured to include both motor and cognitive rehabilitation components, delivered sequentially within the same treatment session. A typical session consisted of:

- Warm-up and positioning exercises (5–10 min).
- Motor rehabilitation activities (20–25 min).
- Cognitive training exercises (15–20 min).
- Cool-down, feedback, and reassessment (5 min).

The 45–60-minute duration represents the total combined time of all therapeutic activities performed within a single session. Individual tasks lasting 10–15 min, as presented in Table 1, refer to subcomponents of this overall session and not separate treatment sessions.

### Progression and individualization

Although weekly therapeutic goals (Week 1–Week 4) were outlined to provide a structured framework, the intervention followed a criterion-based progression model rather than a strictly time-dependent schedule. Advancement to higher-level functional activities was determined by clinical assessment of the participant’s: Motor control, Postural stability, and Cognitive engagement, Fatigue tolerance and Safety considerations. Participants who remained at an early functional stage (e.g., requiring passive range of motion or sitting balance training during Weeks 2 or 3) continued foundational exercises until adequate readiness for progression was achieved. Conversely, participants presenting with mild baseline impairments were progressed more rapidly to advanced task-oriented, balance, coordination, and functional mobility exercises. Early-stage exercises were not unnecessarily prolonged in such cases. Thus, the protocol maintained a standardized structure while allowing individualized adaptation based on recovery status.

### Integration of motor and cognitive components

Both motor and cognitive domains were addressed within each treatment session. The distribution of time allocated to each domain varied according to individual patient needs and therapeutic goals. Cognitive tasks included attention training, memory exercises, problem-solving activities, and orientation tasks integrated with functional movement when feasible.

The reference in Table 1 to “2–4 sessions per week” reflects the scheduling of structured therapeutic focus across the week; however, each 45–60-minute session incorporated multimodal components rather than isolating motor or cognitive training exclusively.

### Repetition and task modification

Session content was partially repeated across treatments in accordance with established principles of motor learning and neuroplasticity, which emphasize repetition, task specificity, and progressive challenge in stroke rehabilitation. While core exercises were reinforced to consolidate skill acquisition, task complexity, intensity, and duration were progressively modified based on individual improvement. All sessions were delivered by trained rehabilitation personnel following a standardized protocol to ensure treatment fidelity while preserving necessary clinical flexibility.

The sessions were administered by licensed physiotherapists with a minimum of three years of clinical experience in stroke rehabilitation. These professionals underwent a two-day orientation and hands-on training program conducted by a senior rehabilitation specialist to ensure consistency in delivering the intervention protocol. Intervention fidelity was monitored using a structured checklist that recorded adherence to session components, duration, patient responsiveness, and any deviations. Weekly supervision meetings were held to review session logs and reinforce protocol adherence.

### Control group care

The control group (*n* = 26) comprised hemiplegic stroke patients who received standard care in accordance with institutional stroke management protocols for a period of four weeks. This routine care focused on maintaining functional stability, preventing secondary complications, and ensuring patient comfort. Physiotherapy was administered twice daily as per physician prescription and included passive range-of-motion exercises, positioning and regular repositioning to prevent pressure ulcers, gentle stretching, and basic mobility facilitation. These sessions focused primarily on motor maintenance rather than structured cognitive rehabilitation. Comprehensive nursing care was uniformly provided and included monitoring of vital signs and neurological status, medication administration, maintenance of skin integrity, assistance with activities of daily living, nutritional support, hygiene care, and prevention of complications. Basic psychological support was offered through therapeutic communication to reduce anxiety and maintain cognitive orientation. Caregivers were educated regarding mobility assistance, feeding techniques, hygiene practices, positioning strategies, and home-based preventive measures. Participants in the control group were not blinded to their treatment; however, they were not informed of the specific research hypothesis or anticipated superiority of any intervention, thereby minimizing expectancy bias. Importantly, both the control and experimental groups received identical standard medical and nursing care throughout the study period. The experimental group received the structured Early Multimodal Rehabilitation (EMR) program in addition to standard care, whereas the control group received standard care alone. No components of the EMR protocol particularly structured cognitive rehabilitation were introduced into the control group. This design ensured that any observed differences in outcomes could be attributed specifically to the EMR intervention rather than differences in routine care or therapeutic exposure.

### Statistical analysis

Data were analyzed using IBM SPSS Statistics version 25.0 (IBM Corp., Armonk, NY, USA). Descriptive statistics, including frequency, percentage, mean, and standard deviation, were used to summarize demographic and clinical characteristics of the participants. Baseline comparability between the experimental and control group was assessed using the Chi-square test for categorical variables and the independent samples t-test for continuous variables. The effectiveness of the intervention over time was evaluated using Repeated Measures Analysis of Variance (RM-ANOVA) to examine the main effects of time, group, and the interaction effect (time × group) on stroke severity, cognitive function, and motor function outcomes. In addition to statistical significance testing, effect size was calculated using partial eta squared (ηp²) to determine the magnitude of the intervention effect. Partial eta squared represents the proportion of total variance in the dependent variable attributable to a specific factor after accounting for other variables in the model. Effect sizes were interpreted using conventional benchmarks: small (ηp² = 0.01), medium (ηp² = 0.06), and large (ηp² ≥ 0.14). A p-value of ≤ 0.05 was considered statistically significant.

**Table 1:** **Early Rehabilitation Protocol Plan for Hemiplegic Stroke Patients** The table-1 outlines the structured weekly rehabilitation program administered over four weeks, consisting of progressive motor and cognitive function activities delivered in multiple sessions per week.

Week & Session	Motor function activities	Cognitive function activities
Week 1: Mobilization & Sensory Stimulation:		
Session − 1	Supported sitting balance training(10 min)	Orientation to personal, spatial, and temporal contexts(10 min)
Session − 2	Position changes (every 2 h) to prevent pressure sores(Throughout the admission period)	Visual and verbal memory recall: Using pictures or flashcards(10 min)
Session-3	Passive range-of-motion exercises (shoulder, elbow, hip, knee)(15 min)	
Session-4	Tactile and proprioceptive stimulation: Massage of the affected limb to enhance sensory input and circulation(15 min)	
Week 2: Introduction of Passive Movements & Cognitive Tasks		
Session-1	Assisted passive movements for the upper and lower limbs(10 min)	Memory training: Familiar face recognition, object recall(15 min)
Session-2	Flexion and extension of major joints (with assistance)(10 min)	Attention exercises: Identifying differences in images or objects(15 min)
Session-3	Bridging exercises (lying supine, lifting hips with support)(10 min)	
Week 3: Progress to Active Movements and Executive Function		
Session- 1	Active-assisted exercises: Adduction/abduction of affected limbs with support (gradually increasing resistance)(15 min)	Simple executive tasks: Following multi-step instructions (e.g., brushing teeth, dressing)(10 min)
Session-2	Strengthening exercises (Thera-band exercises for upper and lower limbs)(10 min)	Simple arithmetic exercises: Counting coins, basic math(10 min)
Session-3	Gait training with parallel bars(15 min)	
Week 4: Advanced Mobility and Complex Problem Solving		
Session − 1	Standing balance exercises (with increasing difficulty, e.g., standing on uneven surfaces)(15 min)	Problem-solving tasks: Planning daily routines, solving puzzles(10 min)
Session-2	Gait training with uneven surfaces and stairs(10 min)	Recall and reasoning exercises: Recalling past events, discussing hypothetical scenarios(10 min)
Session-3	Task-specific training: Walking with supervision, sit-to-stand transfers(15 min)	

The early multimodal rehabilitation protocol was implemented within a nurse-coordinated multidisciplinary framework in the stroke unit. While licensed physiotherapists delivered structured range-of-motion exercises and task-oriented motor retraining sessions, the broader components of the rehabilitation program were primarily administered and reinforced by trained nursing staff. Nurses were responsible for initiating early mobilization outside scheduled physiotherapy sessions, monitoring hemodynamic stability, supervising positioning and postural alignment, facilitating cognitive engagement tasks (including memory recall, orientation exercises, and problem-solving activities), and providing caregiver education to ensure continuity of therapeutic activities. This collaborative model aligns with contemporary stroke care guidelines emphasizing nurse-led coordination of early rehabilitation within multidisciplinary teams^21,22^. Thus, although physiotherapists delivered specific motor-focused components, the multimodal rehabilitation protocol was operationalized and sustained through structured nursing care practices integrated into routine stroke unit management.

## Results

**Table 2:** Socio-demographic profile of both Experimental and control group.

Sample	Characteristics	Control group F (%)	Experimentalgroup F (%)	Chi-square (χ²)	*P*-value
**Age years**	25–40	2(7.7)	7(26.9)	3.92	0.141
	41–56	11(42.3)	12(46.2)		
	57–72	13(50.0)	7(26.9)		
**Gender**	Male	15(57.7)	17(65.4)	0.343	0.558
	Female	11(42.3)	9(34.6)		
**Education**	School	6(23.1)	6(23.1)	0.269	0.874
	Higher secondary	8(30.8)	9(34.6)		
	Graduate & above	12(46.2)	11(42.3)		
**Occupation**	Self-employee	9(34.6)	9(34.6)	0.829	0.661
	Private employee	8(30.8)	11(42.3)		
	Govt employee	9(34.6)	6(23.1)		
**Habits**	smoking	16(61.5)	18(69.2)	1.95	0.376
	Alcohol	5(19.2)	2(7.7)		
	Tobacco	5(19.2)	6(23.1)		
**Co-morbid condition**	Diabetes	2(7.7)	0(0.0)	2.41	0.299
	Blood pressure	13(50.0)	17(65.4)		
	Both blood pressure and diabetes	11(42.3)	9(34.6)		

**Table 3:** Comparison of Mean and SD of Stroke Severity, Cognitive Function, and Motor Function Scores Between Control and Experimental groups at different time points

Outcome Measure	Time Point	Control Group (*n* = 26) Mean ± SD	Experimental Group (*n* = 26) Mean ± SD
**Stroke Severity (NIHSS)**	Baseline	19.00 ± 1.72	18.19 ± 1.78
	Week 2	17.54 ± 2.25	18.80 ± 2.43
	Week 4	13.15 ± 2.22	2.80 ± 1.05
**Cognitive Function (MMSE)**	Baseline	11.00 ± 1.06	11.69 ± 1.69
	Week 2	13.69 ± 1.67	18.54 ± 2.28
	Week 4	18.31 ± 1.85	25.92 ± 1.29
Motor Function (FMA)	Baseline	54.54 ± 4.69	54.23 ± 4.08
	Week 2	59.58 ± 5.02	71.85 ± 4.36
	Week 4	81.00 ± 7.80	92.62 ± 3.36

**Abbreviations**: NIHSS, National Institutes of Health Stroke Scale; MMSE, Mini-Mental State Examination; FMA, Fugl-Meyer Assessment. Stroke severity categories were defined according to Brott et al.^16^; cognitive impairment levels according to Folstein et al.^17^; and motor impairment classifications according to Fugl-Meyer et al.^18^.

**Table 4:** Comparison of Pre-test and Post-test Stroke Severity, Cognitive Function, and Motor Function Scores Between Control and Experimental Groups.

Measure (Scale)	Group (*n* = 26)	Category	Pre-test *n* (%)	Post-test *n* (%)	χ²(df)	*p*-value
**NIHSS (Stroke Severity)**	Control	Minor (< 5)	0 (0.0)	0 (0.0)	χ²(2) = 49.62	< 0.001
		Moderate (5–15)	3 (11.5)	**20 (76.9)**		
		Mod–Severe (16–20)	**23 (88.5)**	6 (23.1)		
		Severe (21–42)	0 (0.0)	0 (0.0)		
	Experimental	Minor (< 5)	0 (0.0)	**24 (92.3)**	χ²(2) = 47.08	< 0.001
		Moderate (5–15)	1 (3.8)	2 (7.7)		
		Mod–Severe (16–20)	**25 (96.2)**	0 (0.0)		
**MMSE (Cognitive Function)**	Control	Normal (24–30)	0 (0.0)	0 (0.0)	χ²(2) = 41.54	< 0.001
		Mild (19–23)	0 (0.0)	11 (42.3)		
		Moderate (10–18)	**26 (100.0)**	15 (57.7)		
	Experimental	Normal (24–30)	0 (0.0)	**21 (80.8)**	χ²(2) = 38.92	< 0.001
		Mild (19–23)	0 (0.0)	5 (19.2)		
		Moderate (10–18)	**26 (100.0)**	0 (0.0)		
**FMA (Motor Function)**	Control	Marked (50–84)	**26 (100.0)**	13 (50.0)	χ²(2) = 28.00	< 0.001
		Moderate (85–94)	0 (0.0)	13 (50.0)		
		Slight (95–99)	0 (0.0)	0 (0.0)		
	Experimental	Marked (50–84)	**26 (100.0)**	0 (0.0)	χ²(2) = 29.54	< 0.001
		Moderate (85–94)	0 (0.0)	14 (53.8)		
		Slight (95–99)	0 (0.0)	**12 (46.2)**		

**Abbreviations**: NIHSS, National Institutes of Health Stroke Scale; MMSE, Mini-Mental State Examination; FMA, Fugl-Meyer Assessment. Stroke severity categories were defined according to Brott et al.^16^; cognitive impairment levels according to Folstein et al.^17^; and motor impairment classifications according to Fugl-Meyer et al.^18^.

The table-4 presents the pre- and post-test distribution of stroke severity (NIHSS), cognitive function (MMSE), and motor function (FMA) in both the control and experimental groups.

For stroke severity (NIHSS), the control group showed a significant shift from moderate to moderate-severe levels, with 88.5% classified as moderate-severe at pre-test, compared to only 23.1% at post-test (χ²(2) = 49.62, *p* < 0.001). The experimental group, however, demonstrated substantial improvement, with 92.3% of patients falling into the minor category post-test compared to 96.2% being in the moderate-severe category pre-test (χ²(2) = 47.08, *p* < 0.001).

In terms of cognitive function (MMSE), the control group showed a significant improvement, with 42.3% of patients shifting from moderate cognitive impairment to mild impairment post-test (χ²(2) = 41.54, *p* < 0.001). The experimental group exhibited remarkable recovery, with 80.8% moving into the normal range (24–30) post-test, compared to no patients in this range pre-test (χ²(2) = 38.92, *p* < 0.001).For motor function (FMA), the control group demonstrated a shift from marked motor impairment (50–84) to moderate impairment (85–94), with 50% of patients showing improvement post-test (χ²(2) = 28.00, *p* < 0.001). The experimental group had a significant improvement as well, with no patients remaining in the marked impairment category post-test and a substantial number (53.8%) improving to moderate impairment and 46.2% reaching slight impairment (χ²(2) = 29.54, *p* < 0.001).Overall, all measures indicate significant improvements in both groups, but the experimental group showed more pronounced recovery, suggesting the effectiveness of the intervention.

**Table 5:** Repeated Measures ANOVA Results for stroke sevirity Over 4 Weeks.

Parameter	Effect	MS (Error)	F (df₁, df₂)	ηp²	*p*-value
Stroke Severity (NIHSS)	Time	1852 (94)	676.9 (3, 48)	0.96	< 0.001
	Group	2175 (100)	169.2 (1, 50)	0.77	< 0.001
	Time×Group	424 (94)	155.2 (3, 48)	0.76	< 0.001

**Abbreviations**: NIHSS, National Institutes of Health Stroke Scale; MS, mean square; df₁, numerator degrees of freedom; df₂, denominator degrees of freedom; F, F-statistic; ηp², partial eta squared; p, probability value.

**Fig. 1:**
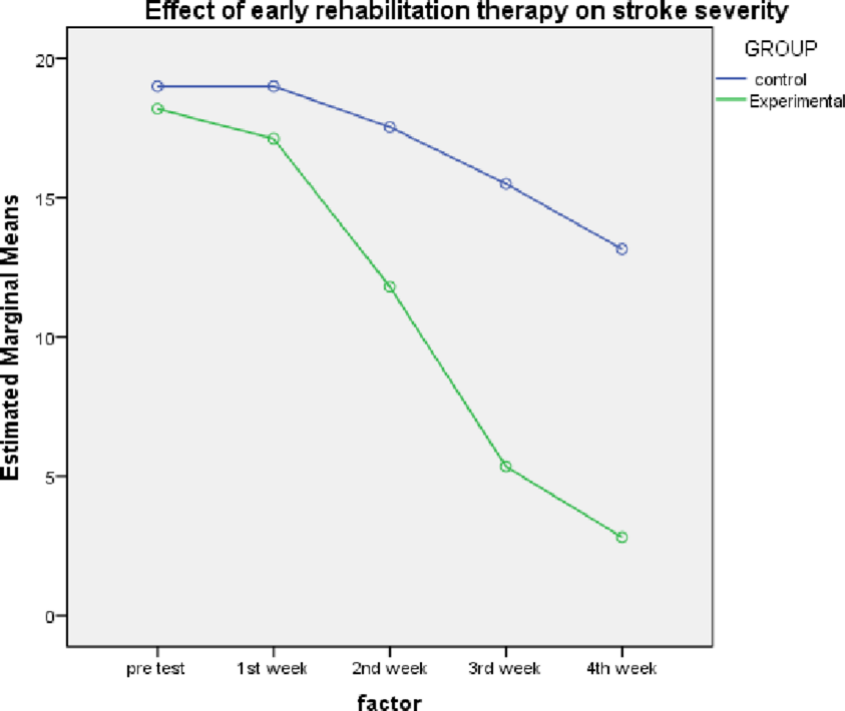
line graph showing effect of early rehabilitation therapy on stroke severity.

The ANOVA for NIHSS scores (Table 5) revealed highly significant main effects of Time (MS = 1852, F(3, 48) = 676.9, ηp² = 0.96, *p* < 0.001) and Group (MS = 2175, F(1, 50) = 169.2, ηp² = 0.77, *p* < 0.001), as well as a significant Time × Group interaction (MS = 424, F(3, 48) = 155.2, ηp² = 0.76, *p* < 0.001). Time effect indicates that, across both groups, stroke severity decreased significantly over the four weeks(Fig 1). Group effect confirms that the experimental group had overall lower (better) NIHSS scores than the control group. The interaction shows that the experimental group improved at a greater rate than controls—clinically, 92.3% of experimental patients shifted to “minor stroke” by week 4 versus none in the control arm. The very large ηp² values (≥ 0.76) reflect a robust impact of the intervention on neurological recovery.

**Table 6:** Repeated Measures ANOVA Results for Cognitive Function Over 4 Weeks.

Parameter	Effect	MS (Error)	F (df₁, df₂)	ηp²	*p*-value
**Cognitive Function (MMSE)**	Time	1424 (45)	767 (3, 48)	0.92	< 0.001
	Group	1232 (46)	123.2 (1, 50)	0.56	< 0.001
	Time × Group	129 (48)	69.8 (3, 48)	0.44	< 0.001

**Abbreviations**: MMSE, Mini-Mental State Examination; MS, mean square; df₁, numerator degrees of freedom; df₂, denominator degrees of freedom; F, F-statistic; ηp², partial eta squared; p, probability value.

**Fig. 2:**
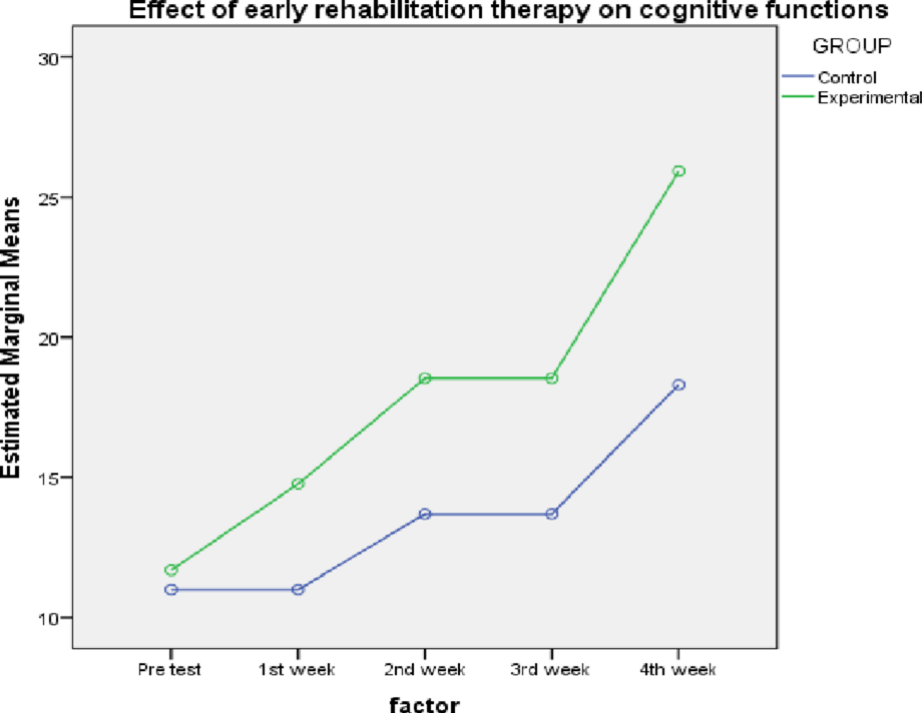
line graph showing effect of early rehabilitation therapy on cognitive functions.

For MMSE scores (Table 6), there were significant effects of Time (MS = 1424, F(3, 48) = 767.0, ηp² = 0.92, *p* < 0.001), Group (MS = 1232, F(1, 50) = 123.2, ηp² = 0.56, *p* < 0.001), and their interaction (MS = 129, F(3, 48) = 69.8, ηp² = 0.44, *p* < 0.001). The Time effect demonstrates substantial overall gains in cognitive scores over time. The Group effect indicates(Fig. 2) that the experimental group achieved higher MMSE scores than the control group on average. The significant interaction reveals that cognitive improvements were far more pronounced in the experimental group, with 80.8% reaching “normal” cognition by week 4 versus none in controls. Partial η² values (0.44–0.92) suggest moderate to large practical significance of early cognitive rehabilitation.

**Table 7:** Repeated Measures ANOVA Results for Motor Function Over 4 Weeks.

Parameter	Effect	MS (Error)	F (df₁, df₂)	ηp²	*p*-value
**Motor Function (Fugl-Meyer)**	Time	24,484 (90)	551 (2, 49)	0.89	< 0.001
	Group	5095 (92)	58.5 (1, 50)	0.53	< 0.001
	Time × Group	1424 (91)	33.8 (2, 49)	0.63	< 0.001

**Abbreviations**: Fugl-Meyer, Fugl-Meyer Assessment; MS, mean square; df₁, numerator degrees of freedom; df₂, denominator degrees of freedom; F, F-statistic; ηp², partial eta squared; p, probability value.

A comparison of socio-demographic characteristics in table-2 between the control (*n* = 26) and experimental (*n* = 26) groups showed no statistically significant differences, confirming successful group matching at baseline. Age distributions across the three brackets (25–40, 41–56, 57–72 years) were similar (control: 7.7%, 42.3%, 50.0%; experimental: 26.9%, 46.2%, 26.9%; χ²(2) = 3.92, *p* = 0.141). Gender proportions did not differ (males: 57.7% vs. 65.4%; χ²(1) = 0.343, *p* = 0.558). Educational attainment was comparable—23.1% in both groups had school-level education, roughly one‐third held higher secondary qualifications, and 42–46% were graduates or above (χ²(2) = 0.269, *p* = 0.874). Occupational status (self-employed, private, government employees) also showed no significant variance (χ²(2) = 0.829, *p* = 0.661). Lifestyle habits of smoking (61.5% vs. 69.2%), alcohol use (19.2% vs. 7.7%), and tobacco use (19.2% vs. 23.1%) were similarly distributed (χ²(2) = 1.95, *p* = 0.376). Finally, the prevalence of co-morbid conditions diabetes only, hypertension only, or both—did not differ significantly (χ²(2) = 2.41, *p* = 0.299). These non-significant chi-square results across all variables ensure that any subsequent differences in clinical outcomes can be attributed to the intervention rather than baseline demographic discrepancies.

## Discussion

The present randomized controlled study evaluated the effectiveness of a structured early multimodal rehabilitation program integrating cognitive stimulation with progressive motor retraining during acute hospitalization for hemiplegic stroke patients. The findings demonstrated statistically significant and clinically meaningful improvements in neurological severity (NIHSS), cognitive function (MMSE), and motor recovery (FMA) in the experimental group compared with standard care. While early rehabilitation has been widely recommended in contemporary stroke guidelines^19–21^, the integration of structured cognitive-motor protocols during the acute neuroplastic window remains insufficiently explored.

### Stroke severity (NIHSS)

The substantial reduction in NIHSS scores (χ²=47.08, *p* < 0.001; ηp²=0.76) supports the growing emphasis on early activation strategies. Recent guidelines emphasize the importance of early structured rehabilitation but primarily focus on mobilization and prevention of complications^21^. Similarly, McDonnell and Hillier^22^ highlighted the neurobiological rationale for early intervention but noted heterogeneity in protocol integration.

Notably, the AVERT-DOSE trial^23^ refined mobilization intensity but did not incorporate structured cognitive engagement. In contrast, our intervention simultaneously stimulated executive and motor networks, potentially enhancing distributed cortical reorganization. Emerging neuroimaging evidence suggests that early multimodal activation enhances structural connectivity restoration across motor and associative networks^24^, supporting the mechanistic plausibility of our findings.

### Cognitive recovery (MMSE)

Cognitive outcomes improved significantly in the experimental group (χ²=38.92, *p* < 0.001). A 2020 Cochrane review confirmed benefits of cognitive rehabilitation post-stroke but emphasized that most interventions were delivered during subacute or chronic phases^25^. Similarly, Barker-Collo et al^26^. reported that early cognitive impairment is prevalent yet often under-addressed during acute rehabilitation.

Few randomized trials have integrated structured cognitive tasks concurrently with motor retraining within the first two weeks post-stroke. Recent experimental models suggest that cognitive-motor coupling enhances task-dependent plasticity and improves dual-task performance^27^. Our findings extend this evidence by demonstrating that early-phase integration is feasible and associated with accelerated cognitive normalization.

### Motor recovery (FMA)

Motor function improved significantly (χ²=29.54, *p* < 0.001; ηp²=0.58), consistent with contemporary systematic reviews emphasizing intensity and task specificity^28^. However, Cirstea and Levin^29^ highlighted that many motor gains in early rehabilitation may reflect compensatory strategies rather than true motor recovery.

The magnitude of FMA improvement observed in our study suggests enhanced restorative recovery, potentially facilitated by simultaneous cognitive engagement improving motor planning and executive control. Koch et al^24^. demonstrated that enhanced structural connectivity in motor networks predicts superior functional recovery, supporting the hypothesis that integrated stimulation may promote adaptive neural reorganization.

### Distinguishing features and novel contribution

While multiple studies have evaluated early mobilization^23,28^ or cognitive rehabilitation independently^25^, the following elements distinguish the present investigation:

- **Simultaneous Cognitive–Motor Integration during Acute Phase**.

Most prior randomized controlled trials investigating early stroke rehabilitation have primarily focused on motor retraining or early mobilization as isolated interventions^23,28^. Although cognitive rehabilitation has independently demonstrated benefits in post-stroke recovery^25^, structured integration of cognitive stimulation with progressive motor training during the acute inpatient phase remains limited. In contrast, the present study implemented a coordinated multimodal protocol within 72 h to 14 days post-stroke, combining executive function tasks, memory engagement, and task-oriented motor exercises in a structured and progressive manner. Emerging evidence suggests that concurrent activation of cognitive and motor circuits enhances network-level plasticity and improves task-dependent cortical reorganization^22,24^. By simultaneously engaging distributed neural systems during the critical early recovery window, this integrated approach may facilitate synergistic improvements beyond what is achieved through single-domain rehabilitation strategies.

b. **Multidomain Outcome Evaluation**.

Another distinguishing feature of this study is the concurrent assessment of neurological severity (NIHSS), cognitive function (MMSE), and motor performance (FMA). Many previous trials have focused predominantly on motor recovery or functional independence outcomes^28^, while cognitive recovery has often been examined separately^25^. By evaluating these domains simultaneously, the present investigation provides a comprehensive recovery profile that captures interconnected pathways of neurological restoration. This multidimensional evaluation allows for a more holistic understanding of stroke recovery dynamics, recognizing that motor, cognitive, and neurological improvements are interdependent rather than isolated phenomena. Such an integrated outcome framework strengthens the interpretability and clinical relevance of the findings.

c. **Early Neuroplastic Window Targeting**.

Experimental and clinical neurorehabilitation research increasingly emphasizes the importance of early intervention during the period of heightened neuroplasticity following stroke^22^. Structural and functional imaging studies indicate that early stimulation enhances synaptic remodeling, promotes network reconnection, and supports adaptive cortical reorganization^24^. However, despite this growing mechanistic understanding, many rehabilitation protocols delay structured cognitive engagement or apply fragmented intervention models. By deliberately targeting the early neuroplastic window with multimodal stimulation, the present study aligns with contemporary network-based models of recovery and provides clinical evidence supporting the translational application of neuroplasticity principles in acute stroke care.

d. **Implementation in a Resource-Constrained Tertiary Setting**.

Most large-scale stroke rehabilitation trials have been conducted in high-income countries with advanced rehabilitation infrastructure and specialized multidisciplinary teams^21,23^. Evidence regarding the feasibility and effectiveness of structured multimodal early rehabilitation protocols in resource-constrained tertiary settings remains comparatively sparse. The present study contributes context-specific evidence demonstrating that an integrated cognitive–motor protocol can be implemented effectively within such healthcare environments. This contextual relevance enhances the generalizability of the findings and supports the scalability of early multimodal rehabilitation strategies in diverse clinical settings where rehabilitation resources may be limited.

Collectively, this study advances current stroke rehabilitation literature by demonstrating that early integrated cognitive-motor rehabilitation yields superior multidimensional recovery compared to conventional care. These findings align with evolving neurorehabilitation paradigms that emphasize network-based recovery models rather than isolated domain-specific interventions^30^.

### Clinical implications and nursing perspective

The present study highlights the central role of nursing care in operationalizing early multimodal rehabilitation during acute stroke recovery. Although physiotherapists delivered structured range-of-motion and task-oriented motor exercises, the comprehensive rehabilitation framework was largely implemented and reinforced by nursing staff. Contemporary stroke management guidelines recognize nurses as primary coordinators of early mobilization, monitoring, and interdisciplinary rehabilitation continuity^21^. Nurses maintain continuous bedside presence, enabling repeated mobilization opportunities, reinforcement of cognitive engagement activities, prevention of secondary complications, and caregiver training throughout hospitalization. Evidence suggests that structured nurse-led early rehabilitation protocols enhance adherence, safety, and functional recovery^22,23^.

Recent rehabilitation models emphasize that recovery optimization depends not solely on isolated therapy sessions but on sustained, repetitive, and contextually embedded stimulation throughout the day^28^. Nurses play a pivotal role in ensuring such distributed therapeutic exposure by integrating cognitive orientation exercises, environmental stimulation, positioning strategies, and assisted functional activities into routine care. Furthermore, early mobilization facilitated by nursing staff has been associated with improved neurological outcomes and reduced complications^23,31^. By embedding cognitive-motor engagement within daily nursing interactions, this study leveraged continuous therapeutic reinforcement rather than limiting recovery stimulation to scheduled physiotherapy sessions.

Importantly, multidisciplinary stroke rehabilitation frameworks increasingly recognize nurse-led coordination as essential for translating neuroplasticity principles into practice^32^. Structural connectivity research indicates that frequent task repetition and multisystem activation promote adaptive neural remodeling^24^, and nurses are uniquely positioned to provide this repeated stimulation across the continuum of inpatient care. Therefore, while physiotherapists contributed essential motor-specific expertise, the sustained multimodal engagement delivered through structured nursing care likely played a substantial role in the multidimensional improvements observed. Critical care nurses play a pivotal role in the early identification of patients suitable for rehabilitation and in facilitating early mobilization and cognitive stimulation. Training nurses in the principles of neurorehabilitation can help ensure timely intervention, reduce the risk of complications, and improve overall stroke recovery outcomes. This study emphasizes the need for a collaborative, nurse-led approach to rehabilitation planning and delivery in acute care units.

### Study strength, limitations and future directions

A major strength of this study lies in its structured intervention design and use of validated outcome tools (NIHSS, MMSE, FMA). Additionally, real-time monitoring of intervention fidelity ensured consistent delivery. Although the results are promising, the study has several limitations. The quasi-experimental design and small sample size may limit the generalizability of the findings. Future randomized controlled trials with larger sample sizes are needed to validate these results and assess the long-term effects of early rehabilitation therapy. Additionally, further research should explore the most effective components of rehabilitation (e.g., the optimal timing, intensity, and combination of therapies) to maximize recovery in stroke patients. A limitation of the present study is that detailed clinical variables such as precise stroke onset duration and lesion laterality were not systematically recorded. Future research should incorporate these parameters to allow more comprehensive baseline characterization and exploration of their potential influence on rehabilitation outcomes.

## Conclusion

In conclusion, the findings of this study support the efficacy of early rehabilitation therapy in improving stroke severity, cognitive function, and motor function in hemiplegic stroke patients. The experimental group showed significantly greater improvements in all three domains, emphasizing the importance of early intervention in optimizing recovery outcomes. The integration of cognitive and motor rehabilitation, combined with a structured, multidisciplinary approach, can significantly enhance post-stroke recovery. Future research should continue to explore the long-term effects of early rehabilitation and identify the most effective rehabilitation strategies for stroke patients.

Table 3: At baseline, both groups were comparable in stroke severity (NIHSS: 19.00 ± 1.72 vs. 18.19 ± 1.78), cognitive function (MMSE: 11.00 ± 1.06 vs. 11.69 ± 1.69), and motor function (FMA: 54.54 ± 4.69 vs. 54.23 ± 4.08), indicating group homogeneity (*p* > 0.05). By Week 4, the experimental group showed significantly greater improvement compared to the control group, with a marked reduction in NIHSS scores (2.80 ± 1.05 vs. 13.15 ± 2.22), and higher gains in MMSE (25.92 ± 1.29 vs. 18.31 ± 1.85) and FMA scores (92.62 ± 3.36 vs. 81.00 ± 7.80). Repeated-measures ANOVA revealed a statistically significant time × group interaction effect for all outcome measures (*p* < 0.001), indicating that early multimodal rehabilitation was more effective than conventional care in improving neurological severity, cognitive function, and motor recovery over the four-week period.

**Fig. 3:**
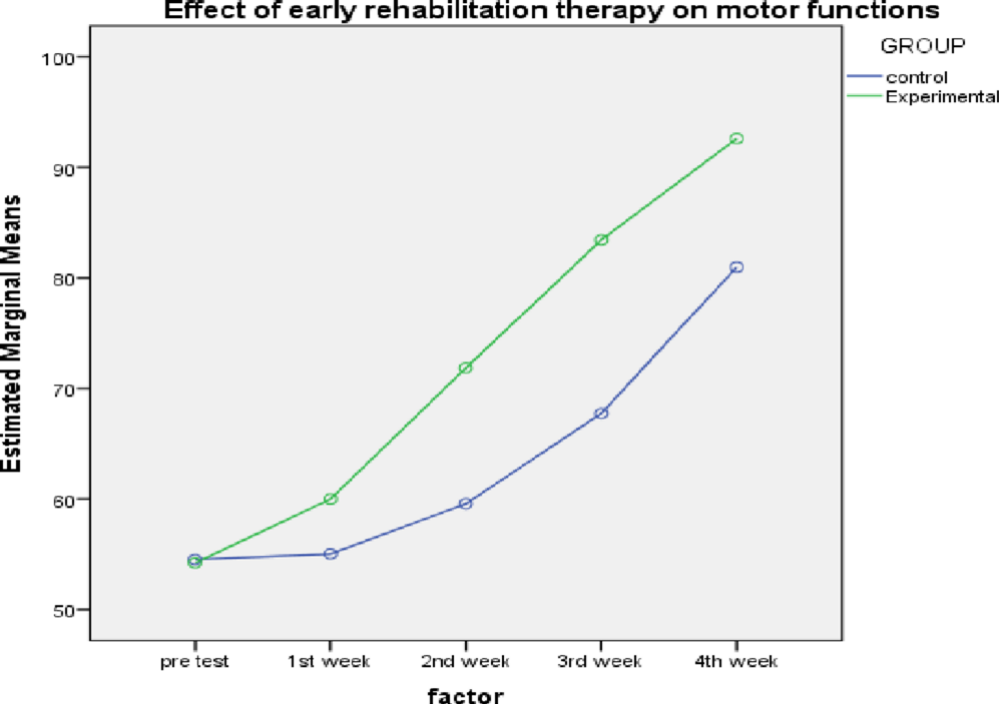
line graph showing effect of early rehabilitation therapy on motor functions.

Table 7 shows significant Time (MS = 24 484, F(2, 49) = 551.0, ηp² = 0.96, *p* < 0.001), Group (MS = 5095, F(1, 50) = 58.5, ηp² = 0.53, *p* < 0.001), and Time × Group interaction (MS = 1424, F(2, 49) = 33.8, ηp² = 0.58, *p* < 0.001). The Time effect confirms significant motor recovery across all patients. The Group effect shows that those receiving early rehabilitation consistently outperformed controls in FMA scores. The significant interaction indicates(Fig-3) that the experimental group’s motor improvements outpaced the control group’s—by week 4, nearly half (46.2%) of experimental patients achieved only slight dyscoordination, whereas no control patients did. Effect sizes (ηp² ≥ 0.53) underscore a strong intervention effect on motor outcomes. Across all three domains neurological severity, cognition, and motor function early rehabilitation produced large, clinically meaningful improvements that progressed more rapidly and to a greater extent than standard care alone. The significant interactions in each ANOVA confirm that the experimental group not only improved over time but did so at a significantly greater rate, validating the efficacy of structured early intervention in hemiplegic stroke recovery.

## Data Availability

The datasets generated and analyzed during the current study are not publicly available due to privacy and ethical restrictions but are available from the corresponding author upon reasonable request.
